# 38% silver diamine fluoride for dentine hypersensitivity treatment: an *in situ* study

**DOI:** 10.3389/froh.2026.1862698

**Published:** 2026-06-04

**Authors:** Dhananthat Chawhuaveang, Alice Kit Ying Chan, Chun Hung Chu, Ollie Yiru Yu

**Affiliations:** 1 Department of Pediatric Dentistry, Faculty of Dentistry, Thammasat University, Pathum Thani, Thailand; 2 Faculty of Dentistry, The University of Hong Kong, Hong Kong SAR, China

**Keywords:** dental erosion, dentine, dentine hypersensitivity, remineralization, silver diamine fluoride

## Abstract

**Introduction and aim:**

Silver diamine fluoride (SDF) is a desensitizing agent approved by the U.S. Food and Drug Administration. However, limited evidence is available to guide the optimal application protocol for SDF. This *in situ* study aimed to compare dentinal tubule occlusion scores and examine the elemental composition of occluding materials after single versus weekly applications of 38% SDF over 2 weeks.

**Methods:**

Five healthy participants took part in this *in situ* study, which included three 14-day experimental periods and a 7-day washout between periods. In the first experimental period, distilled water (DI) was applied on Day 1. In the second experimental period, SDF was applied once on Day 1. In the third experimental period, SDF was applied on Days 1 and 8. Each participant wore an intraoral palatal appliance containing four treated dentine blocks during the experimental periods. The dentine blocks were removed from the participants’ mouths and exposed extraorally to citric acid (pH 3.2) for 2 min, five times daily, for 14 days. Dentinal tubule occlusion scores and the elemental composition of the occluding materials were evaluated using scanning electron microscopy (SEM) images and energy-dispersive x-ray spectroscopy (EDS), respectively. Dentinal tubule occlusion scores were analyzed using one-way analysis of variance (ANOVA) followed by Tukey's HSD *post hoc* test, whereas EDS elemental composition data were analyzed using the Kruskal–Wallis test followed by Dunn's *post hoc* test with Bonferroni correction.

**Results:**

All five participants completed all three experimental periods. The mean dentinal tubule occlusion scores were 1.80 ± 0.35 for the DI group, 3.29 ± 0.27 for the single-application SDF group, and 4.10 ± 0.10 for the weekly-application SDF group (DI < single-application SDF < weekly-application SDF; *p* < 0.001). EDS analysis of dentine blocks revealed a silver peak in both SDF groups, with a higher silver weight percentage in the weekly-application SDF group than in the single-application SDF and DI groups (DI < single-application SDF < weekly-application SDF; *p* < 0.001).

**Conclusion:**

Although not without limitations, this study reveals that a weekly protocol results in superior dentinal tubule occlusion scores compared to a single application, and that increased frequency of SDF application directly contributes to a higher weight percentage of silver in the resulting occluding materials.

## 1 Introduction

Dentine hypersensitivity is characterized by short, sharp pain arising from exposed dentine in response to external stimuli (1). The primary cause of dentine hypersensitivity is dentine exposure (2), which may result from gingival recession, dental caries, overzealous toothbrushing, or tooth wear (1, 2). The hydrodynamic theory is the most widely accepted explanation of the mechanism underlying dentine hypersensitivity (1). When dentine is exposed, fluid movement within the dentinal tubules can be induced by thermal, tactile, chemical, osmotic, or electrical stimuli (1, 3). These stimuli cause movement of dentinal fluid within the tubules, triggering mechanoreceptors and resulting in pain associated with dentine hypersensitivity (1). Consequently, dentine hypersensitivity can significantly affect quality of life by interfering with speaking and eating (4, 5).

Dentine hypersensitivity is a common clinical condition in the general population (3). A recent systematic review reported that the estimated prevalence of dentine hypersensitivity was 32% among young and older adults (3). Dentine hypersensitivity in these groups is associated with several oral conditions, including dental caries, periodontal disease, tooth wear, and non-carious cervical lesions, all of which can expose the dentine surface and contribute to dentine hypersensitivity (3, 6, 7). The systematic review and meta-analysis by Zeola et al. reported that young adults had a higher prevalence of dentine hypersensitivity than other age groups (6). This higher prevalence may be partly explained by habits and lifestyle factors that increase young adults’ exposure to acidic foods and beverages, which may promote dentine hypersensitivity (6, 8). However, dentine hypersensitivity symptoms in children and young adults may be reduced by obliteration of dentinal tubules and the formation of secondary and tertiary dentine (6).

Older adults are particularly susceptible to dentine hypersensitivity because of age-related physiological changes in the gingiva and tooth surface (1). Older adults with gingival recession and dentine exposure often experience dentine hypersensitivity following periodontal treatment (3, 5). A previous systematic review reported that approximately three-quarters of patients who underwent periodontal treatment experienced dentine hypersensitivity (9). In addition, severe tooth wear becomes more common with advancing age (3). These factors may contribute substantially to dentine hypersensitivity in older adults (3, 5). Additionally, older adults may perceive and express pain differently than younger individuals (10). Therefore, managing dentine hypersensitivity in older adults can be complex and challenging (1). Older adults often have limited physical function and reduced manual dexterity, which may interfere with restorative procedures or advanced dental treatments (1). Furthermore, older adults with cognitive impairments may have difficulty following instructions for using at-home desensitizing agents (1, 11).

Controlling dentine hypersensitivity is generally challenging. In addition to patient education for managing risk factors for dentine exposure (4, 5), desensitizing agents are commonly used to alleviate dentine hypersensitivity during symptomatic episodes (1, 5, 12). Desensitizing agents are commonly classified into two types: those that promote dentinal tubule occlusion and those that reduce neural transmission (13). Sodium fluoride, calcium carbonate, and arginine are commonly used dentinal tubule-occluding agents (13, 14). These agents primarily deposit material within dentinal tubules on exposed dentine surfaces, thereby decreasing dentine permeability (13). Potassium nitrate is a commonly used nerve-desensitizing agent that is thought to reduce neural excitability and dentine sensitivity (13, 14). Both types of desensitizing agents can be used either at home or in office-based clinical settings (15). At-home desensitizing agents are generally considered a first-line treatment option for dentine hypersensitivity (15). However, at-home desensitizing agents often have limited bioavailability and require a high level of patient adherence, which may lead to unsatisfactory results (15). Although many commercially available products are marketed for dentine hypersensitivity treatment (13, 15), no single treatment is considered to be universally accepted, and the clinical evidence supporting desensitizing agents remains limited (1, 15). Professionally applied desensitizing treatments may be more suitable for older adults and children, who often require safe, simple procedures that reduce treatment complexity and chairside time (1). Desensitizing agents with sustained effects should therefore be prioritized for the treatment of dentine hypersensitivity in these populations (14).

Silver diamine fluoride (SDF) is a desensitizing agent approved by the U.S. Food and Drug Administration (FDA) (16). The main active components of SDF are silver and fluoride ions (4, 16). Silver ions interact with exposed collagen fibrils to form silver-protein complexes, whereas fluoride ions contribute to the formation of precipitated mineral layers on the treated surface (12, 17, 18). Previous laboratory studies have investigated the penetration depth of silver and fluoride ions from SDF into dentine, with reported values ranging from 40 to 1,200 μm (2, 19–22). These reactions may occlude exposed dentinal tubules, thereby decreasing dentine permeability and reducing dentinal fluid movement (23). Previous clinical studies have investigated the effect of SDF on reducing dentine hypersensitivity (7, 12, 24, 25). The studies showed that SDF may alleviate dentine hypersensitivity by lowering pain scores, such as visual analogue scale (VAS) or sensitivity scores (SSs). However, despite these improvements, symptoms of dentine hypersensitivity still persisted (12, 24, 25).

Previous studies have evaluated weekly SDF application protocols in dental erosion and dentine caries research, reporting superior anti-erosive protection and improved dentine caries arrest compared with less frequent application protocols (26–28). Nevertheless, the effect of different SDF application frequencies on dentinal tubule occlusion remains unclear. Dentinal tubule occlusion is considered one mechanism through which desensitizing agents may mitigate dentine hypersensitivity. Moreover, an *in situ* design is widely used in dental research because it allows experimental conditions to be evaluated in the oral environment while minimizing risk to participants, thereby providing greater clinical relevance than laboratory studies alone (29). Therefore, this *in situ* study aimed to evaluate the effect of single versus weekly applications of 38% SDF on dentinal tubule occlusion and the elemental composition of occluding materials. The null hypothesis was that different application frequencies of 38% SDF would have no effect on dentinal tubule occlusion or the elemental composition of occluding materials.

## 2 Materials and methods

### 2.1 Ethical approval

This study was conducted in accordance with the Declaration of Helsinki and the Guidelines for Good Clinical Practice after the study protocol was approved by the Institutional Review Board (IRB) of the University of Hong Kong/Hospital Authority, Hong Kong West Cluster (IRB reference: UW 20–210).

### 2.2 Study design

**Figure 1:**
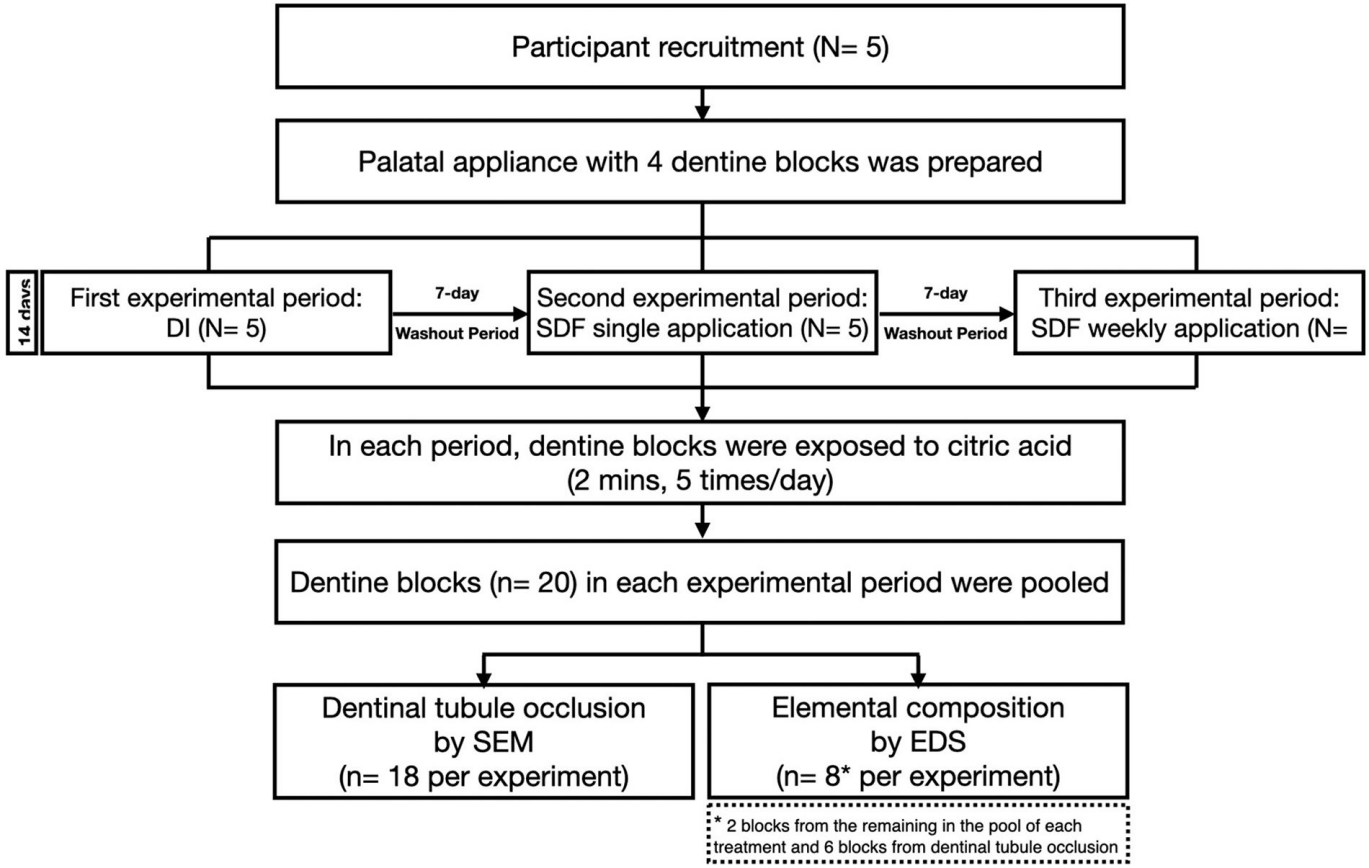
Flowchart of the study. N, number of participants; n, number of dentine blocks; SDF, silver diamine fluoride; DI, distilled water; SEM, scanning electron microscopy; EDS, energy-dispersive x-ray spectroscopy.

This study was designed as a crossover *in situ* study with three 14-day experimental periods and a 7-day washout period between periods. Accordingly, the three experimental periods consisted of application of distilled water (DI), a single application of silver diamine fluoride (SDF), and weekly applications of SDF over 14 days. The study flowchart is shown in Figure 1.

In the first experimental period, DI served as the negative control and was applied to each dentine block on Day 1 using a microbrush for 1 min before air-drying for 1 min. In the second and third experimental periods, SDF was applied at different frequencies. Five microliters of 38% SDF solution (Advantage Arrest, Elevate Oral Care, West Palm Beach, FL, USA) were applied to the dentine blocks using a microbrush for 1 min, and the blocks were then air-dried for 1 min, following the manufacturer's instructions (12, 30). The second experimental period consisted of a single SDF application on Day 1. The third experimental period consisted of weekly SDF application, with SDF applied on Days 1 and 8.

### 2.3 Sample size calculation

This dentinal tubule occlusion score was identified as a key indicator of the potential desensitizing effect of SDF across different application protocols. Sample size calculation was performed using G*Power software, version 3.1. The sample size was determined based on a previous study that investigated dentinal tubule occlusion after treatment with various fluoride agents (31). The calculation used an effect size of 0.68, a power of 0.90, and a significance level of 0.05. The computed sample size was 33 dentine blocks for analysis of the dentinal tubule occlusion score (11 per group). Ultimately, 18 dentine blocks per group were included to ensure adequate statistical power.

### 2.4 Participant recruitment

The inclusion criteria were agreement with the study procedures and purpose, age of at least 18 years, good general health with a normal salivary flow rate, and absence of active caries or periodontitis. The exclusion criteria were allergy to fluoride, silver, or dental materials; use of orthodontic appliances or removable prostheses; pregnancy or breastfeeding; and use of medications that might affect salivary flow rate. Based on the required sample size of 18 dentine blocks per group for analysis of the dentinal tubule occlusion score and the initially planned sample of 2 dentine blocks per group for analysis of the elemental composition of the occluding materials, five participants were recruited. Each participant received intraoral palatal appliances containing four dentine blocks during each experimental period. The mean age of the three female and two male participants was 30 ± 3 years.

### 2.5 Dentine block preparation

Twenty molars were collected from patients who required tooth extraction after informed consent was obtained. Extracted teeth were cleaned and stored in a 0.1% thymol solution. Each extracted molar was cut to obtain a 2-mm-thick dentine slice; in total, 20 dentine slices were prepared. The dentine slices were polished using 2,000-grit and 4,000-grit sandpaper. Each dentine slice was then cut into three dentine blocks (3 mm in width, 3 mm in length, and 2 mm in thickness), which were allocated across the three groups. After that, each dentine slice was cut into three dentine blocks (3 mm in width, 3 mm in length, and 2 mm in thickness), which were allocated to three groups. The dentine blocks were cleaned with 18% EDTA (Sigma-Aldrich, St. Louis, MO, USA) for 10 s and then ultrasonicated twice in deionized water for 5 min each. The dentine blocks were then examined under a stereomicroscope at 40 × magnification to verify the presence of open dentinal tubules and ensure that no smear layer remained. This confirmed dentine exposure, representing a model of a hypersensitive surface. Dentine blocks with defects were discarded. The dentine blocks were disinfected by immersion in 70% ethanol for 30 min (32, 33).

### 2.6 Intraoral appliance preparation

**Figure 2:**
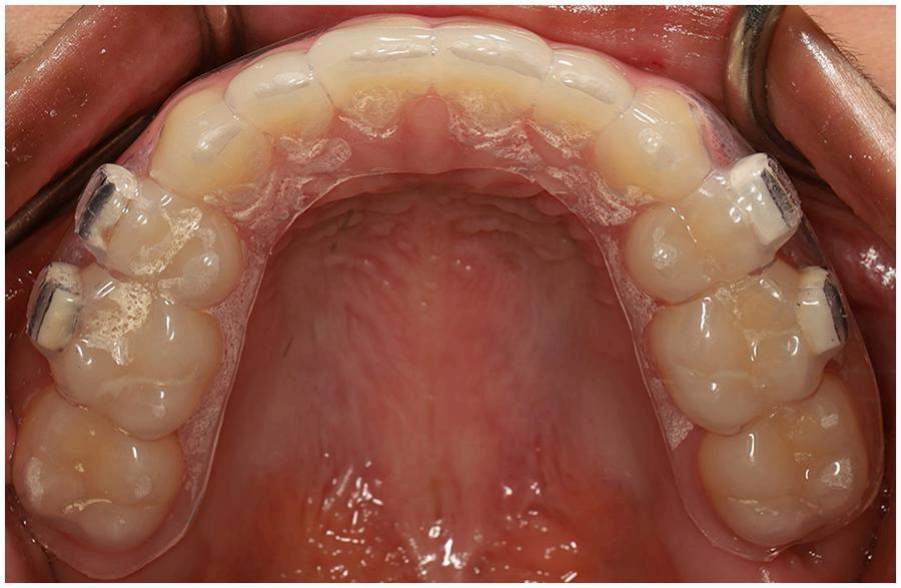
A representative palatal intraoral appliance.

All participants underwent scaling and polishing before impressions were taken for fabrication of the intraoral appliances. Maxillary impressions were obtained using alginate material and subsequently cast with dental stone. A palatal intraoral appliance was fabricated using a 1.35-mm-thick, soft dental splint sheet (HMT Splint Sheet, Henan Smart Industrial, Henan, China). Four dentine blocks, two on each side, were fixed to the buccal surface of the appliance in the second premolar and first molar regions. All blocks were placed approximately 0.5 mm below the outer surface of the appliance to protect them from friction and abrasive forces. A representative palatal intraoral appliance used in this study is shown in Figure 2. Because this study included three groups, each participant was provided with three palatal intraoral appliances.

### 2.7 *In situ* experiment

The researchers provided standardized oral hygiene tools, including fluoridated toothpaste (Colgate Total Original®, Colgate-Palmolive, New York, NY, USA), a soft manual toothbrush (Systema Toothbrush Standard Soft, Lion Corporation, Bangkok, Thailand), and dental floss (Oral-B Essential Floss, Procter & Gamble, Cincinnati, OH, USA). Participants were instructed to use these oral hygiene tools for 7 days before the experiment, throughout each experimental period, and during the washout periods. Participants were not allowed to use any other oral hygiene products. Written instructions and a procedure schedule were provided to promote adherence to the study protocol. Participants were thoroughly instructed in all procedures before the experiment began and were required to record completion of all study procedures during the study.

Each treatment period started with treatment of the dentine block surfaces. The intraoral appliances carrying treated dentine blocks were worn for 1 h before the first acidic challenge. The intraoral appliances were immersed extraorally in 50 mL of citric acid (pH 3.2; Sigma-Aldrich, St. Louis, MO, USA) for 2 min and then thoroughly rinsed in distilled water for 2 min. Afterward, the intraoral appliances were reinserted into the participants’ mouths. The acidic challenge was repeated five times daily at 1-h intervals for 14 days. These procedures were designed to simulate dietary acid exposure in the oral environment and assess the acid resistance of the treated dentine surfaces.

Participants were instructed to refrain from consuming food while wearing the appliances, although they were allowed to sip water. Participants were advised to remove the intraoral appliances for 1 h during lunch. Participants were permitted to clean the appliances at the end of each day. The intraoral appliances were stored overnight in moist gauze inside a plastic container.

### 2.8 Dentinal tubule occlusion measurement

Eighteen dentine blocks from each treatment group were evaluated for dentinal tubule occlusion. Dentine blocks were ultrasonicated twice in deionized water for 5 min each and immersed in a 2% glutaraldehyde solution at 4 °C overnight. After fixation, dentine blocks were dehydrated in different concentrations of ethanol (50%, 70%, 85%, 95%, and 100%) for 10 min each and were critical-point dried using a critical point dryer (Leica EM CPD300, Leica Microsystems, Germany) overnight. The dentine blocks were mounted on an aluminum plate and sputter-coated with carbon (Quorum Q150T ES Plus, Quorum Technologies Ltd, Laughton, UK). Each dentine block was examined for dentinal tubule occlusion using a scanning electron microscope (Hitachi S-4800 FEG Scanning Electron Microscope, Hitachi Ltd., Tokyo, Japan) at an accelerating voltage of 5 kV and a magnification of 5,000 ×.

**Figure 3:**
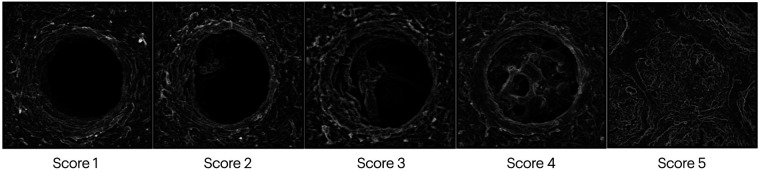
Dentinal tubule occlusion levels based on a 5-point visual scoring index.

Dentine blocks were examined using SEM and images were captured. Images were captured at the center of the dentine blocks (31). The level of dentinal tubule occlusion was evaluated using a 5-point visual scoring index, as shown in Figure 3. A 5-point visual score index was modified from previous studies (8, 31, 34). Score 1 represented entirely open dentinal tubules, score 2 represented predominantly open dentinal tubules, score 3 represented an equal proportion of occluded and open dentinal tubules, score 4 represented predominantly occluded dentinal tubules, and score 5 represented complete dentinal tubule occlusion. All SEM images of the dentine blocks from all experimental groups were numbered with Arabic numerals and then randomized before assessment. The dentinal tubule occlusion level for each SEM image was scored by one dentist, blinded to group allocation. Fifty-four SEM images were re-examined by the same examiner to assess intra-examiner reliability for dentinal tubule occlusion scoring. The mean dentinal tubule occlusion score was determined by averaging the respective values of representative SEM images of each treatment.

### 2.9 Elemental composition assessment

Although two dentine blocks per group were initially planned for EDS analysis, six additional dentine blocks per group were evaluated after dentinal tubule occlusion assessment to strengthen the elemental composition analysis. Elemental composition within the occluded dentinal tubules was quantified using energy-dispersive x-ray spectroscopy (EDS) with an X-Max 80 EDS detector and AZtec software (Oxford Instruments, High Wycombe, UK). Area-scan elemental composition analysis was performed at an accelerating voltage of 5 kV and a magnification of 500 ×. The EDS analysis identified calcium (Ca), phosphorus (P), fluorine (F), chlorine (Cl), and silver (Ag), and their quantities were expressed as weight percent (wt%).

### 2.10 Statistical analysis

All analyses were conducted using IBM SPSS Statistics, version 27.0 for Windows (IBM Corp., Armonk, NY, USA). The significance level was set at *p* < 0.05 and values of *p* < 0.05 were considered statistically significant. The Shapiro–Wilk test was used to evaluate data normality and Levene's test was used to assess homogeneity of variance. A one-way analysis of variance (ANOVA), followed by Tukey's HSD *post hoc* test, was performed to compare dentinal tubule occlusion scores among the three experimental groups. Because the EDS elemental composition data were not normally distributed and did not meet the assumption of homogeneity of variance, the Kruskal–Wallis test was used, followed by Dunn's *post hoc* test with Bonferroni correction for pairwise comparisons.

## 3 Results

### 3.1 Dentinal tubule occlusion

**Figure 4:**
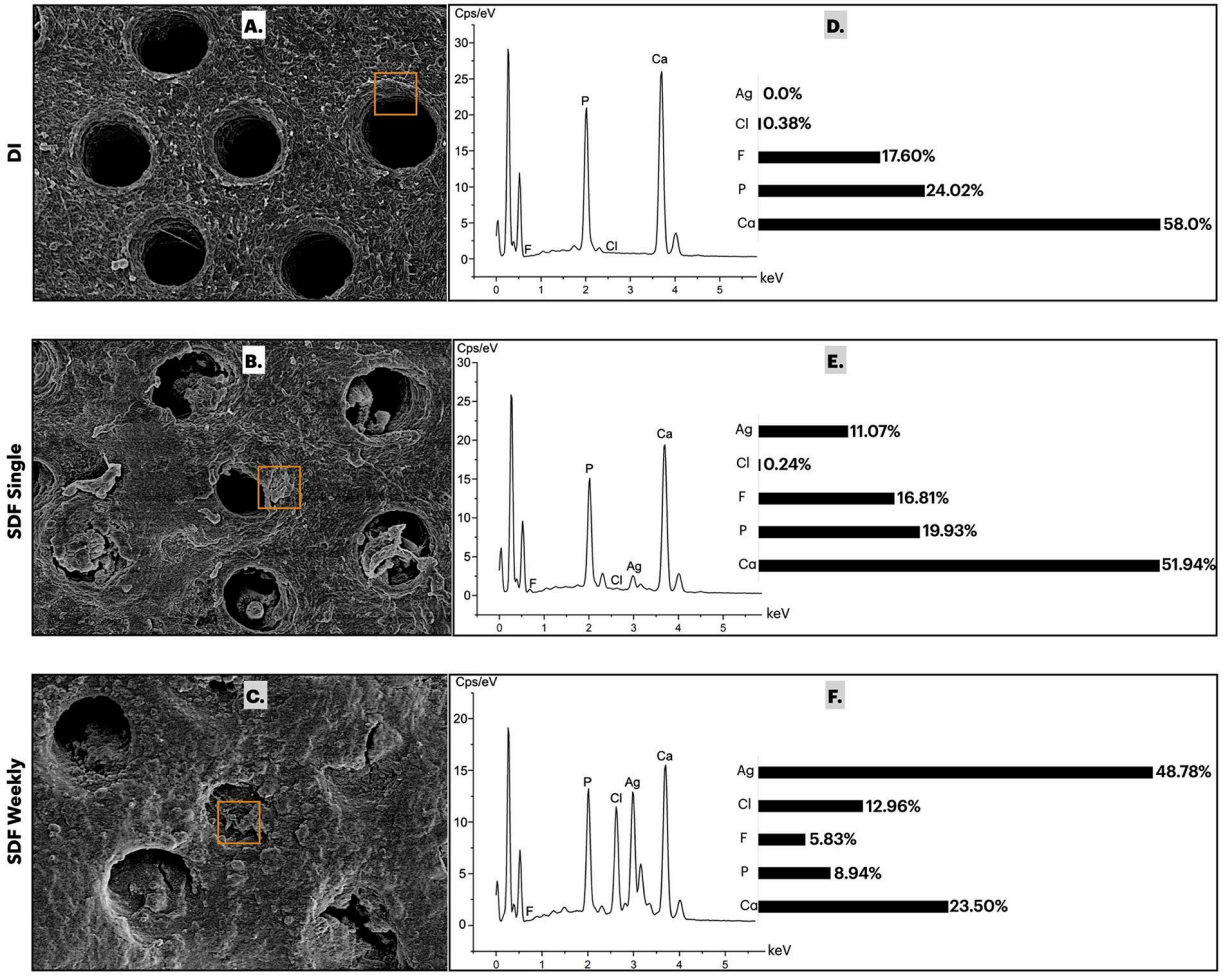
**(A–C)** depict SEM images of the dentine surfaces in the three groups at a magnification of 5,000 ×, and **(D–F)** present the EDS analysis of the elemental composition of the occluding materials within the dentinal tubules. SDF, silver diamine fluoride; DI, distilled water; Ag, silver; Cl, chlorine; F, fluorine; P, phosphorus; Ca, calcium.

Figures 4A–C show SEM images of the dentine surfaces in the three groups. Intra-examiner reliability was assessed using the intraclass correlation coefficient (ICC). The ICC for dentinal tubule occlusion scoring was 0.80. The mean ± SD dentinal tubule occlusion scores were 1.80 ± 0.35 for the DI group, 3.29 ± 0.27 for the single-application SDF group, and 4.10 ± 0.10 for the weekly-application SDF group. Statistically significant differences were observed among the three groups (*p* < 0.001). The dentinal tubule occlusion score for the DI group was significantly lower than those for the single-application SDF and weekly-application SDF groups (*p* < 0.001 for both comparisons). Furthermore, the dentinal tubule occlusion score for the weekly-application SDF group was significantly higher than that for the single-application SDF group (*p* < 0.001).

### 3.2 Elemental composition

Figures 4D–F and Table 1 show the weight percentages of elements in the occluding materials within the dentinal tubules of the three groups, as determined by EDS. Peaks for calcium (Ca), phosphorus (P), chlorine (Cl), and fluorine (F) were observed in all groups. A silver (Ag) peak was detected in the occluding materials within the dentinal tubules of dentine surfaces treated with both the single-application and weekly-application SDF protocols (Figures 4E,F). The wt% of Ag and Cl was significantly higher in the weekly-application SDF group than in the single-application SDF and DI groups (*p* < 0.001). The wt% of F in the weekly-application SDF group was significantly lower than in the single-application SDF and DI groups (*p* < 0.001).

**Table 1:** Weight percentages (wt%) of elements in the occluding materials within the dentinal tubules of the three groups.

	elemental composition				
Group	Ag	Cl	F	P	Ca
DI	0.00 ^A^	0.38 ^A^	17.60 ^A^	24.02 ^A^	58.0 ^A^
SDF single	11.07 ^B^	0.24 ^A^	16.81^A^	19.93 ^A,B^	51.94 ^A^
SDF weekly	48.78 ^C^	12.96 ^B^	5.83 ^B^	8.94 ^B^	23.50^B^

Different superscript uppercase letters indicate significant differences among the three groups within each element. SDF, silver diamine fluoride; DI, distilled water; Ag, silver; Cl, chlorine; F, fluorine; P, phosphorus; Ca, calcium.

## 4 Discussion

This study was the first *in situ* investigation to evaluate the effects of different application frequencies of 38% SDF on dentinal tubule occlusion and the elemental composition of occluding materials, using single and weekly applications of SDF over a 2-week period. Weekly application of SDF resulted in a higher dentinal tubule occlusion score and a greater weight percentage of silver ions in the occluding materials. Consequently, the null hypothesis, which stated that different application frequencies of 38% SDF would have no effect on dentinal tubule occlusion or the elemental composition of occluding materials, was rejected. These findings may serve as a basis for future clinical research evaluating whether increased SDF application frequency improves dentine hypersensitivity outcomes. Increasing the frequency of SDF application may enhance dentinal tubule occlusion, which could inform future clinical studies of SDF protocols for dentine hypersensitivity. Moreover, if future clinical studies confirm that increased SDF application frequency improves dentine hypersensitivity outcomes, this approach could offer clinical benefits. Such an approach may be particularly relevant for future studies involving vulnerable patients, such as older adults with physical or cognitive impairments. It may also be relevant to future research involving children with dentine hypersensitivity related to early childhood caries. Additionally, this approach may warrant further investigation in patients with persistent dentine hypersensitivity after unsuccessful at-home desensitizing treatment. It should be noted that the results of this *in situ* study were based on only five participants. Therefore, translation of these findings into clinical practice should be approached with caution.

The mean dentinal tubule occlusion score for dentine surfaces treated with weekly SDF application after a 14-day acidic challenge was significantly higher than that for surfaces treated with single SDF application. In this study, dentinal tubule occlusion was associated with the deposition of silver-containing materials on the treated dentine surfaces, as indicated by SEM and EDS analyses. The SEM images suggested that silver-containing deposits were present not only within patent dentinal tubules but also on dentine surfaces, including peritubular and intertubular dentine (2). SDF application could deliver silver and fluoride ions to dentine surfaces (27). This effect may be attributed to the interaction of silver ions with proteins and collagen fibrils within the dentine matrix, resulting in the formation of silver–protein complexes and silver-containing compounds (4, 35, 36). Additionally, fluoride ions can react with hydroxyapatite and mineral ions present in saliva to form mineral deposits on dentine surfaces (17, 35). Increasing the frequency of SDF application may have led to greater accumulation of these deposits (27), thereby increasing dentinal tubule occlusion. An increased frequency of SDF application may have resupplied greater amounts of silver and fluoride ions to demineralized dentine surfaces (27). This was supported by EDS analysis, which indicated a greater amount of silver in the occluding particles on dentine surfaces treated with weekly SDF application than on those treated with a single SDF application.

The *in situ* conditions may have contributed to dentinal tubule occlusion in SDF-treated dentine. Saliva is a crucial factor in facilitating the reaction of SDF with dentine surfaces (17). Saliva serves as a reservoir of mineral ions, such as calcium and phosphate, that are supplied by the salivary glands (37). Calcium and phosphate ions in saliva can promote apatite formation (17, 38). SDF can modify dentine hydroxyapatite through the substitution of hydroxyl groups, resulting in fluorapatite formation (17). Additionally, SDF can react with hydroxyapatite and saliva to form silver-containing deposits on treated dentine (18, 27). Consequently, dentinal tubule occlusion by these deposits may play a role in reducing dentine hypersensitivity (1, 12). Moreover, silver-containing and other occluding particles within the dentinal tubules may serve as a reservoir, gradually releasing silver and fluoride ions over time (23). These ions may interact with mineral ions present in saliva and on the dentine surface, thereby facilitating the continued formation of apatite (17).

An acidic challenge was conducted to simulate exposure to acidic foods and beverages after SDF application. Citric acid is commonly present in acidic foods and beverages and is frequently used in erosion research to simulate dietary acidic exposure (8, 39). The pH of the citric acid solution used in this study was 3.2, a value commonly used in *in vitro* and *in situ* erosion studies (40, 41). Furthermore, there is no consensus regarding the frequency of acid exposure; in this study, acidic challenges were conducted five times daily to represent three main meals and two snack periods between meals. This acid exposure may have reduced the stability of the SDF-derived deposits throughout the experiment. Such an acidic challenge could lead to dentine demineralization by dissolving dentine hydroxyapatite and the precipitated layer formed after SDF application, including occluding particles within the dentinal tubules. This interpretation was supported by the finding that dentine surfaces treated with a single SDF application and exposed to five acidic challenges daily for 14 days had a dentinal tubule occlusion score of 3.29, indicating an equal proportion of occluded and open dentinal tubules. These findings suggest that the stability of SDF-derived deposits may decrease gradually after application under repeated acidic exposure. However, further studies are needed to verify the acid resistance of the occluding particles formed within the dentinal tubules after SDF application. These findings may help guide postoperative instructions for patients receiving SDF treatment for dentine hypersensitivity; specifically, patients may be advised to limit acidic foods and beverages to help maintain the effectiveness of SDF.

Although this study confirmed that the dentinal tubule occlusion after SDF application increased with higher application frequency, its limitations mean that the findings may not fully reflect clinical anti-hypersensitivity effects. This study employed a crossover *in situ* design; therefore, some procedures could not fully replicate the clinical oral environment, including salivary protein dynamics, pellicle formation, biofilm development, and other intraoral factors. Furthermore, because of its *in situ* design, this study did not evaluate direct clinical outcomes of dentine hypersensitivity, which are typically assessed in response to stimuli such as compressed cold air or water from a triple syringe applied to the hypersensitive surface (7, 12, 30). Common scoring systems for assessing dentine hypersensitivity in randomized controlled trials, such as VAS sensitivity scores ranging from 0 (no pain) to 10 (extreme pain), depend on individuals’ pain perception and pain thresholds and could not be applied in this *in situ* study (7, 12, 30). Alternatively, previous studies of other experimental agents have used dentinal tubule occlusion to evaluate the potential efficacy of desensitizing agents for reducing dentine hypersensitivity in both *in vitro* and *in vivo* settings (8, 31, 42). Dentinal tubule occlusion was therefore considered a suitable surrogate outcome for assessing the potential effect of SDF on dentine hypersensitivity in this *in situ* study. However, dentinal tubule occlusion may not necessarily correspond to clinical effectiveness in alleviating dentine hypersensitivity. In addition, this study examined the elemental composition of occluding materials using EDS. EDS is considered a semi-quantitative technique for elemental analysis. Further investigations using quantitative elemental analysis are needed. Lastly, this study was conducted over a relatively short 14-day period; therefore, future randomized clinical trials with longer follow-up periods and patient-reported hypersensitivity outcomes are needed to determine whether different SDF application protocols provide clinically meaningful reductions in dentine hypersensitivity.

## 5 Conclusion

The findings of this study show that weekly application of SDF increased dentinal tubule occlusion scores and resulted in a higher percentage of silver ions in the occluding material compared with a single application after a 14-day acidic challenge. Within the limitations of this *in situ* study, increasing the frequency of SDF application may improve dentinal tubule occlusion; however, its clinical effectiveness in managing dentine hypersensitivity should be validated in future clinical studies.

## Data Availability

The raw data supporting the conclusions of this article will be made available by the authors, without undue reservation.
